# Development and validation of a predictive model for white matter lesions in young- and middle-aged people

**DOI:** 10.3389/fneur.2023.1257795

**Published:** 2023-10-19

**Authors:** Renwei Zhang, Li Peng, Qi Cai, Yao Xu, Zhenxing Liu, Yumin Liu

**Affiliations:** ^1^Department of Neurology, Zhongnan Hospital of Wuhan University, Wuhan, China; ^2^Department of Cardiology, Zhongnan Hospital of Wuhan University, Wuhan, China; ^3^Department of Neurology, Yiling Hospital of Yichang, Yichang, China

**Keywords:** white matter lesions, carotid plaque score, diastolic blood pressure, metabolic syndrome, nomogram

## Abstract

**Background:**

White matter lesion (WML) is an age-related disorder associated with stroke and cognitive impairment. This study aimed to investigate the risk factors and build a predictive model of WML in young- and middle-aged people.

**Methods:**

We performed a second analysis of the data from the Dryad Digital Repository. We selected those people who are <60 years old and randomly divided them into the training group and the validation group. We investigated the risk factors of WML in the training group with logistic regression analysis and built a prediction nomogram based on multivariate logistic regression analysis; finally, the performance of the prediction nomogram was evaluated for discrimination, accuracy, and clinical utility.

**Results:**

There were 308 people in the training group and 723 people in the validation group. Multivariate regression analysis showed that the age (OR = 1.49, 95% CI: 1.31–1.70), diastolic blood pressure (OR = 1.02, 95% CI: 1.00–1.03), carotid plaque score (OR = 1.31, 95% CI: 1.14–1.50), female gender (OR = 2.27, 95% CI: 1.56–3.30), and metabolic syndrome (OR = 2.12, 95% CI: 1.22–3.70) were significantly associated with white matter lesions. The area under the curve value (AUC) of the receiver operating curve (ROC) was 0.734 for the training group and 0.642 for the validation group. The calibration curve and clinical impact curve showed that the prediction nomogram has good accuracy and clinical application value.

**Conclusion:**

Age, diastolic blood pressure, carotid plaque score, female gender, and metabolic syndrome were risk factors in young- and middle-aged people <60 years old with WML, and the nomogram based on these risk factors showed good discrimination, accuracy, and clinical utility.

## Introduction

The prevalence of WML is high in certain populations, and it increases with age. Nearly 50% of people may suffer some grade of WML at the age of 40 years, with approximately 90% detectable in the general population of people over the age of 60 years (1, 2). WML of presumed vascular origin is frequently seen as hyperintense on T2-weighted images (T2WI) and fluid-attenuated inversion recovery sequence (FLAIR) and hypointense or isointense on T1-weighted images (T2WI) on brain MRI (3). However, the mechanism of WML is incompletely understood, and the pathophysiological mechanisms, such as hypoxia-ischemia, endothelial dysfunction, blood–brain barrier disruption, and infiltration of inflammatory mediators or cytokines, might have an important effect on WML occurrence and development (4).

WML increases the risk level of mild cognitive impairment, dementia, ischemic stroke, and even death (5–8). However, its significance in asymptomatic healthy populations remains poorly defined. Some researchers have found several risk factors related to WML, such as age, female gender, high blood pressure, diabetes mellitus, hyperlipidemia, smoking status, and drinking habit (9, 10). Previous studies have (11–13) analyzed some risk factors of WML in people over 60 years old. However, there are few studies on WML and its risk factors in young- and middle-aged people under 60 years old. Effective identification and improvement of individuals' risk factors for WML in young- and middle-aged people could reduce the occurrence and development of WML and then reduce the risk of stroke and cognitive impairment caused by WML. It is valuable to investigate WML and its risk factors in young- and middle-aged people and build a predictive model.

Based on research on real-world populations, we aimed to build a clinical predictive model of WML in young- and middle-aged people under 60 years old, aiming at early detection of high-risk groups of WML and early intervention.

## Methods and materials

### Data resource

All the data were obtained from the Dryad Digital Repository. Dryad is an open data publishing platform and a community committed to the open availability and routine re-use of all research data. According to Dryad Terms of Service, we cited the Dryad data package (Data from Mathematical modeling for the prediction of cerebral white matter lesions based on clinical examination data, Dryad, Dataset, https://doi.org/10.5061/dryad.73bh2q8) in our study.

### Data collection and study design

**Figure 1:**
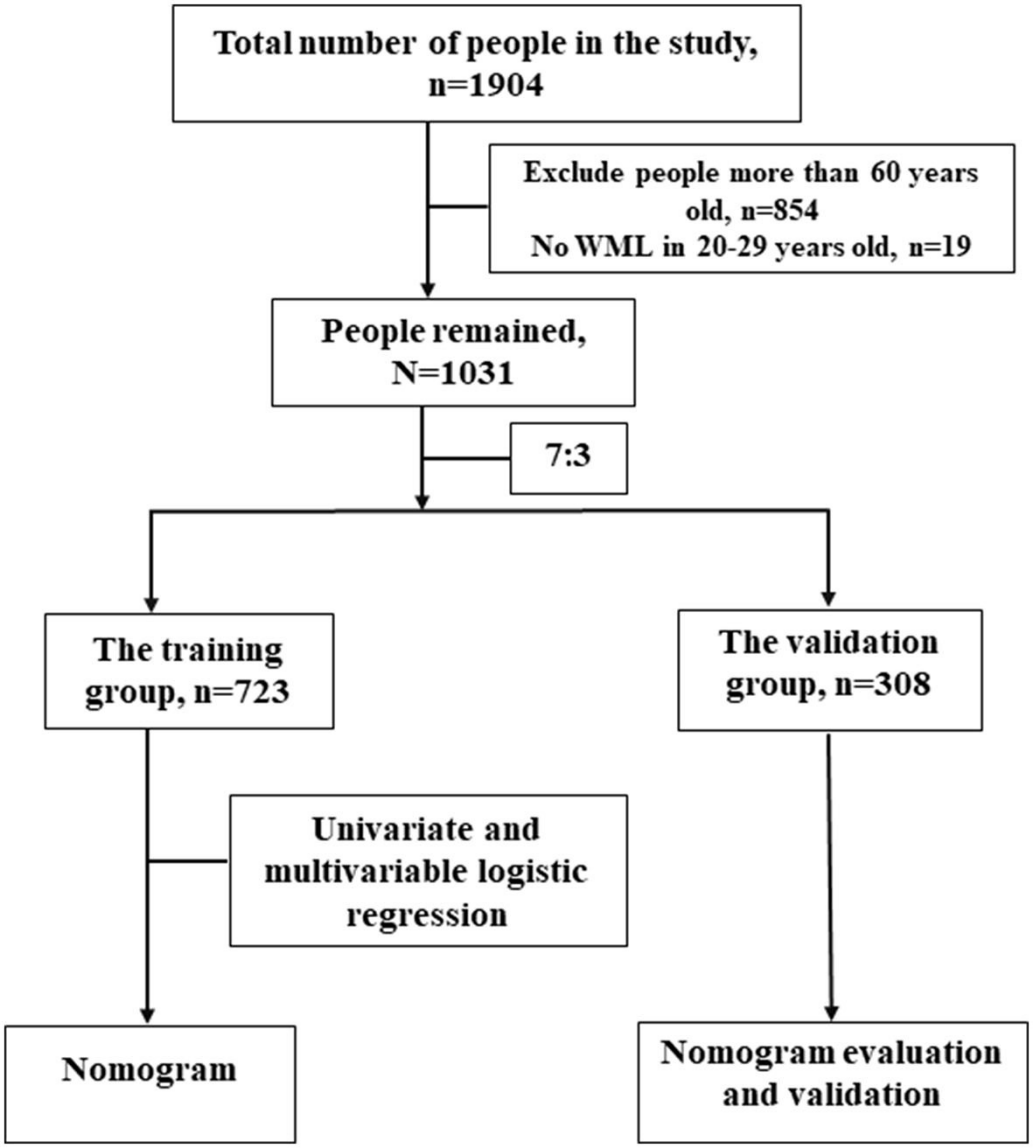
Flowchart of the study.

Our study was performed according to Shinkawa et al. (14) findings. Their research was based on a retrospective study to build a prediction model about WML in 1,904 adults (988 male and 916 female patients) from a comprehensive medical checkup between 1 April 2016 and 31 October 2017 in a Japanese Hospital, where they got head MRI, blood tests, carotid ultrasonography, and completed standard questionnaires. We selected these adults <60 years old and collected data from the comprehensive medical checkup (Figure 1).

Six basic characteristics were obtained during a general inspection, including gender, age, diastolic blood pressure (DBP), body mass index (BMI), systolic blood pressure (SBP), and visceral obesity. The metabolic syndrome was determined by the presence of visceral obesity. The blood and biochemical tests comprised low-density lipoprotein (LDL) cholesterol, high-density lipoprotein cholesterol (HDL), LH ratio (LDL/HDL), hemoglobin A1c (HbA1c), triglyceride (TG), and fasting blood glucose level (BS). The ultrasonic testing recorded carotid plaque score (PS), and the number of plaque and a sum of thicknesses of plaques (intima-media thickness > 1.1 mm) were used to calculate PS from four sections of the carotid artery: the central side of the common carotid artery (CCA), the peripheral side of the CCA, the bifurcation of the CCA, and the central side of the internal carotid artery. A questionnaire about specific health examinations was recorded including medications to reduce blood pressure (Red_bp_med), medications to reduce blood sugar or insulin injection (Insulin), and medications to decrease the level of cholesterol (Red_cho_med). We also collected data about smoking status and drinking alcohol habits (in terms of Sake, a kind of Japanese wine, according to the frequency of drinking, rarely drink, sometimes, every day) and drinking volume (four levels according to the amount of drinking, <180 ml, 180–360 ml, 360–540 ml, and >540 ml per day).

All the subjects underwent head MRI examinations in Shin Takeo Hospital, and T1-weighted images (T1WI), T2-weighted images (T2WI), and fluid-attenuated inversion recovery images (T2FLAIR) were got and analyzed. Based on the results of the image profile, we divided these people into the WML group and the non-WML group.

### Statistical analysis

All statistical analyses were performed with version R 4.1.3 (www.r-project.org). If continuous variables were normally distributed, they were presented as mean ± standard deviation (SD), and if they were not normally distributed, they were shown as median [interquartile range (IQR)]. Categorical variables were described as the number of events and percentages. We analyzed comparisons between continuous variables by Student's *t*-test or the Wilcoxon rank-sum test, and categorical variables were analyzed with the chi-square test or Fisher's exact test, as appropriate. It was deemed statistically significant when a two-sided *p* < 0.05. The R package “compareGroups” was used in these analyses.

Variables with a *p* < 0.05 in the univariate analysis were included in the multivariate logistic regression model, followed by the backward stepwise selection method. By combining the final significant predictors, we established a nomogram to predict the risk probability of the WML. The nomogram's performance was measured by area under the receiver operating characteristic curve (AUC) and assessed by comparing nomogram predicted vs. observed incidences of the outcomes (calibration curve) in both training set and validation set. Furthermore, decision curve analysis (DCA) was conducted to evaluate the clinical utility of the model. The AUC, calibration curve, and DCA were delineated based on regression analysis. R packages “rms”, “pROC”, and “rmda” were used in these analyses.

## Results

### Clinical features and baseline characteristics

A total of 1,031 adults <60 years old were collected in our study, and they were randomly divided into the training group and the validation group according to 7:3. The baseline information of the training group and the validation group is presented in Table 1. No significant difference was found in the baseline information of the two groups. The baseline information of the WML group and the non-WML group from the training group is presented in Table 2. There were 255 people in the WML group and 468 people in the non-WML group. In the general inspection, age in the non-WML group was relatively younger compared with the WML group, and there were more female patients in the WML group than in the non-WML group (50.59 vs. 40.60%, *p* < 0.05). SBP and DBP were higher in the WML group than in the non-WML group. A significant difference was found in the proportion of metabolic syndrome in the two groups (*p* < 0.05), but no significant difference was found in BMI. In the blood biochemical examination, there were significant differences in HbA1c and LDL between the non-WML group and the WML group (p < 0.05). In the specific health examination questionnaire, Red_bp_med and Red_cho_med showed significant differences between the non-WML group and the WML group. In the ultrasonic tests, significant differences were found in the PS and the number of plaques between the non-WML group and the WML group. It was interesting that there was no significant difference in living habits such as smoking and drinking habits between the two groups.

**Table 1:** Baseline characteristics of the training group and validation group.

**Characteristics**	**All (*n =* 1,031)**	**Training group (*n =* 723)**	**Validation group (*n =* 308)**	***p*-value**
PS, mean (SD)	0.56 (1.34)	0.58 (1.38)	0.51 (1.22)	0.4
**Age**, ***n*** **(%)**				0.489
30–34	43 (4.17%)	28 (3.87%)	15 (4.87%)	
35–39	104 (10.09%)	74 (10.24%)	30 (9.74%)	
40–44	177 (17.17%)	134 (18.53%)	43 (13.96%)	
45–49	207 (20.08%)	138 (19.09%)	69 (22.40%)	
50–54	262 (25.41%)	182 (25.17%)	80 (25.97%)	
55–59	238 (23.08%)	167 (23.10%)	71 (23.05%)	
Every increase of 5 years	4.22 (1.44)	4.21 (1.44)	4.24 (1.45)	0.739
BMI, mean (SD), kg/m^2^	23.28 (3.51)	23.27 (3.56)	23.29 (3.39)	0.945
Female, *n* (%)	444 (43.06%)	319(44.12%)	125(41.58%)	0.327
**Metabolic syndrome**, ***n*** **(%)**				0.559
No	799 (77.50%)	566 (78.28%)	233 (75.65%)	
Reserve	108 (10.48%)	75 (10.37%)	33 (10.71%)	
Yes	124 (12.03%)	82 (11.34%)	42 (13.64%)	
**Smoking habit**, ***n*** **(%)**				0.187
No	791 (76.72%)	546 (75.52%)	245 (79.55%)	
Yes	240 (23.28%)	177 (24.48%)	63 (20.45%)	
**Drinking habit**, ***n*** **(%)**				0.525
Rarely	359 (34.82%)	255 (35.27%)	104 (33.77%)	
Sometimes	352 (34.14%)	239 (33.06%)	113 (36.69%)	
Everyday	320 (31.04%)	229 (31.67%)	91 (29.55%)	
**Drink_qt**, ***n*** **(%)**				0.592
< 180 ml	574 (55.67%)	411 (56.85%)	163 (52.92%)	
180–360 ml	301 (29.19%)	208 (28.77%)	93 (30.19%)	
360–540 ml	112 (10.86%)	76 (10.51%)	36(11.69%)	
>540 ml	44 (4.27%)	28 (3.87%)	16 (5.19%)	
**Insulin**, ***n*** **(%)**				0.403
No	995 (96.51%)	695 (96.13%)	300 (97.40%)	
Yes	36 (3.49%)	28 (3.87%)	8 (2.60%)	
LDL-C, mean (SD), mg/dl	120.61 (30.73)	120.89 (30.95)	119.94 (30.23)	0.645
HDL-C, mean (SD), mg/dl	60.25 (15.63)	59.30 (14.87)	61.19 (15.92)	0.388
LH (LDL/HDL), mean (SD)	2.15 (0.81)	2.16 (0.81)	2.12 (0.79)	0.436
Triglycerides, median [IQR], mg/dl	88.00 [61.00; 137.00]	87.00 [61.50; 137.00]	91.00 [60.00; 136.25]	0.767
HbA1c, mean (SD), %	5.65 (0.60)	5.65 (0.60)	5.67 (0.73)	0.705
BS, mean (SD), mg/dl	102.58 (18.46)	102.38 (18.02)	103.04 (19.49)	0.613
SBP, mean (SD), mmHg	120.16 (16.98)	120.17 (17.05)	120.15 (16.84)	0.983
DBP, mean (SD), mmHg	74.14 (12.58)	74.08 (12.79)	74.28 (12.11)	0.811
**Red_bp_med**, ***n*** **(%)**				1.00
No	896 (86.91%)	628 (86.86%)	268 (87.01%)	
Yes	135 (13.09%)	95 (13.14%)	40 (12.99%)	
**Red_cho_med**, ***n*** **(%)**				0.519
No	933 (90.49%)	651 (90.04%)	282 (91.56%)	
Yes	98 (9.51%)	72 (9.96%)	26 (8.44%)	
**WML**, ***n*** **(%)**				1.00
No	668 (64.79%)	468 (64.73%)	200 (64.94%)	
Yes	363 (35.21%)	255 (35.27%)	108 (35.06%)	
**No. of plaque**, ***n*** **(%)**				0.342
0	808 (78.37%)	560 (77.46%)	248 (80.52%)	
1	141 (13.68%)	104 (14.38%)	37 (12.01%)	
2	60 (5.82%)	44 (6.09%)	16 (5.19%)	
3	15 (1.45%)	8 (1.11%)	7 (2.27%)	
4	4 (0.39%)	4 (0.55%)	0 (0.00%)	
5	3 (0.29%)	3 (0.41%)	0 (0.00%)	

DBP, diastolic blood pressure; BMI, body mass index; SBP, systolic blood pressure; LDL-C, low-density lipoprotein cholesterol; HDL-C, high-density lipoprotein cholesterol; BS, blood glucose level; PS, plaque score; Red_bp_med, medication to reduce blood pressure; Insulin, medication to reduce blood sugar or insulin injection; Red_cho_med, medication to reduce a level of cholesterol; Drink_qt, amount of drinking per day; WML, white matter lesion.

**Table 2:** Baseline characteristics of the WML group and the non-WML group in the training group.

	**Total**	**Non-WML group**	**WML group**	***p*-value**
	***N** =* **723**	***N** =* **468**	***N** =* **255**	
PS, mean (SD)	0.58 (1.38)	0.35 (0.93)	0.99 (1.89)	< 0.001
**Age (years):**				< 0.001
30–34	28 (3.87%)	22 (4.70%)	6 (2.35%)	
35–39	74 (10.24%)	66 (14.10%)	8 (3.14%)	
40–44	134 (18.53%)	111 (23.72%)	23 (9.02%)	
45–49	138 (19.09%)	91 (19.44%)	47 (18.43%)	
50–54	182 (25.17%)	101 (21.58%)	81 (31.76%)	
55–59	167 (23.10%)	77 (16.45%)	90 (35.29%)	
Age every increase of 5 years:	4.21 (1.44)	3.88 (1.45)	4.80 (1.23)	< 0.001
BMI, mean (SD), kg/m^2^	23.27 (3.56)	23.15 (3.58)	23.51 (3.54)	0.199
LDL (mg/dl), mean (SD), mg/d	120.89 (30.95)	119.07 (31.91)	124.24 (28.88)	0.027
HDL (mg/dl), mean (SD), mg/d	59.97 (15.26)	59.30 (14.87)	61.19 (15.92)	0.119
LH(LDL/HDL)	2.16 (0.81)	2.15 (0.81)	2.19 (0.83)	0.522
Triglycerides, median [IQR], mg/dl	87.00 [61.50;137.00]	85.50 [62.00;130.00]	92.00 [60.50;149.50]	0.347
HbA1c, mean (SD), %	5.65 (0.60)	5.60 (0.52)	5.74 (0.71)	0.004
BS (mg/dl), mean (SD), mg/dl	102.38 (18.02)	101.58 (16.56)	103.85 (20.38)	0.129
SBP (mmHg), mean (SD), mmHg	120.17 (17.05)	118.65 (15.90)	122.96 (18.68)	0.002
DBP (mmHg), mean (SD), mmHg	74.08 (12.79)	72.77 (11.76)	76.48 (14.19)	0.001
**No. of plaque:**				< 0.001
0	560 (77.46%)	393 (83.97%)	167 (65.49%)	
1	104 (14.38%)	53 (11.32%)	51 (20.00%)	
2	44 (6.09%)	18 (3.85%)	26 (10.20%)	
3	8 (1.11%)	3 (0.64%)	5 (1.96%)	
4	4 (0.55%)	1 (0.21%)	3 (1.18%)	
5	3 (0.41%)	0 (0.00%)	3 (1.18%)	
**Gender:**				0.012
Male	404 (55.88%)	278 (59.40%)	126 (49.41%)	
Female	319 (44.12%)	190 (40.60%)	129 (50.59%)	
**Smoking:**				0.072
No	546 (75.52%)	343 (73.29%)	203 (79.61%)	
Yes	177 (24.48%)	125 (26.71%)	52 (20.39%)	
**Red_bp_med:**				< 0.001
No	628 (86.86%)	425 (90.81%)	203 (79.61%)	
Yes	95 (13.14%)	43 (9.19%)	52 (20.39%)	
**Insulin:**				0.144
No	695 (96.13%)	454 (97.01%)	241 (94.51%)	
Yes	28 (3.87%)	14 (2.99%)	14 (5.49%)	
**Red_cho_med:**				< 0.001
No	651 (90.04%)	437 (93.38%)	214 (83.92%)	
Yes	72 (9.96%)	31 (6.62%)	41 (16.08%)	
**Drink_qt:**				0.746
< 180 ml	411 (56.85%)	261 (55.77%)	150 (58.82%)	
180–360 ml	208 (28.77%)	135 (28.85%)	73 (28.63%)	
360–540 ml	76 (10.51%)	53 (11.32%)	23 (9.02%)	
>540 ml	28 (3.87%)	19 (4.06%)	9 (3.53%)	
**Metabolic syndrome:**				0.003
No	566 (78.28%)	382 (81.62%)	184 (72.16%)	
Reserve	75 (10.37%)	46 (9.83%)	29 (11.37%)	
Yes	82 (11.34%)	40 (8.55%)	42 (16.47%)	
**Drink habit:**				0.86
Rarely drink	255 (35.27%)	163 (34.83%)	92 (36.08%)	
Sometimes	239 (33.06%)	158 (33.76%)	81 (31.76%)	
Everyday	229 (31.67%)	147 (31.41%)	82 (32.16%)	

DBP, diastolic blood pressure; BMI, body mass index; SBP, systolic blood pressure; LDL-C, low-density lipoprotein cholesterol; HDL-C, high-density lipoprotein cholesterol; BS, blood glucose level; PS, plaque score; Red_bp_med, medication to reduce blood pressure; Insulin, medication to reduce blood sugar or insulin injection; Red_cho_med, medication to reduce a level of cholesterol; Drink_qt, amount of drinking per day; WML, white matter lesion.

### Identification of risk factors for WML

The univariate analysis identified the following factors to be associated with WML: PS, age, LDL, HbA1c, SBP, DBP, gender, Red_bp_med, Red_cho_med, and metabolic syndrome in the training group (Table 3). The stepwise backward logistic regression analysis revealed five independent risk factors of WML: age (OR = 1.49, 95% CI: 1.31–1.70), DBP (OR = 1.02, 95% CI: 1.00–1.03), PS (OR = 1.31, 95% CI: 1.14–1.50), female gender (OR = 2.27, 95% CI: 1.56–3.30), and metabolic syndrome (OR = 2.12, 95% CI: 1.22–3.70) (Table 3).

**Table 3:** Identification of risk factors for WML.

	**Univariate logistic regression**			**Multivariate logistic regression**		
	**OR**	**95% CI**	***p*-value**	**OR**	**95% CI**	***p*-value**
**Age**						
30–34	Ref.	Ref.	Ref.			
35–39	0.44	0.14–1.42	0.172			
40–44	0.76	0.28–2.08	0.593			
45–49	1.89	0.72–4.99	0.196			
50–54	2.94	1.14–7.59	0.026			
55–59	4.29	1.65–11.11	0.003			
**PS**	1.43	1.26–1.63	< 0.001	1.31	1.14–1.5	< 0.001
LDL	1.01	1.00–1.01	0.032			
HDL	1.01	1.00–1.02	0.112			
LH	1.06	0.88–1.28	0.518			
TG	1	1.00–1.00	0.969			
HbA1c	1.5	1.15–1.95	0.003			
BS	1.01	1.00–1.02	0.112			
SBP	1.01	1.01–1.02	0.001			
DBP	1.02	1.01–1.04	< 0.001	1.02	1–1.03	0.028
**Number of plaque:**						
0	Ref.	Ref.	Ref.			
1	2.26	1.48–3.46	< 0.001			
2	3.4	1.81–6.37	< 0.001.			
3	3.92	0.93–16.6	0.063			
4	7.06	0.73–68.37	0.092			
5	4984699	0-Inf	0.976			
BMI	1.03	0.99–1.07	0.2			
**Gender:**						
Male	Ref.	Ref.	Ref.			
Female	1.5	1.10–2.04	0.01	2.27	1.56–3.3	< 0.001
**Smoking habit:**						
No	Ref.	Ref.	Ref.			
Yes	0.7	0.48–1.01	0.058			
**Red_bp_med:**						
No	Ref.	Ref.	Ref.			
Yes	2.53	1.63–3.93	< 0.001			
**Insulin:**						
No	Ref.	Ref.	Ref.			
Yes	1.88	0.87–4.07	0.107			
**Red_cho_med:**						
No	Ref.	Ref.	Ref.			
Yes	2.69	1.64–4.45	< 0.001			
**Drink_qt:**						
< 180 ml	Ref.	Ref.	Ref.			
180–360 ml	0.94	0.66–1.33	0.735			
360–540 ml	0.76	0.44–1.27	0.301			
>540 ml	0.83	0.35–1.85	0.659			
Age every increase of 5 years:	1.65	1.45–1.86	< 0.001	1.49	1.31–1.7	< 0.001
**Metabolic syndrome:**						
No	Ref.	Ref.	Ref.			
Reserve	1.31	0.79–2.15	0.292			
Yes	2.18	1.36–3.49	0.001	2.12	1.22–3.7	0.008
**Drink habit:**						
Rarely drink	Ref.	Ref.	Ref.			
Sometimes	0.91	0.63–1.32	0.612			
Everyday	0.99	0.68–1.43	0.951			

DBP, diastolic blood pressure; BMI, body mass index; SBP, systolic blood pressure; LDL-C, low-density lipoprotein cholesterol; HDL-C, high-density lipoprotein cholesterol; BS, blood glucose level; PS, plaque score; Red_bp_med, medication to reduce blood pressure; Insulin, medication to reduce blood sugar or insulin injection; Red_cho_med, medication to reduce a level of cholesterol; Drink_qt, amount of drinking per day; WML, white matter lesion.

### Development of the clinical prediction model

**Figure 2:**
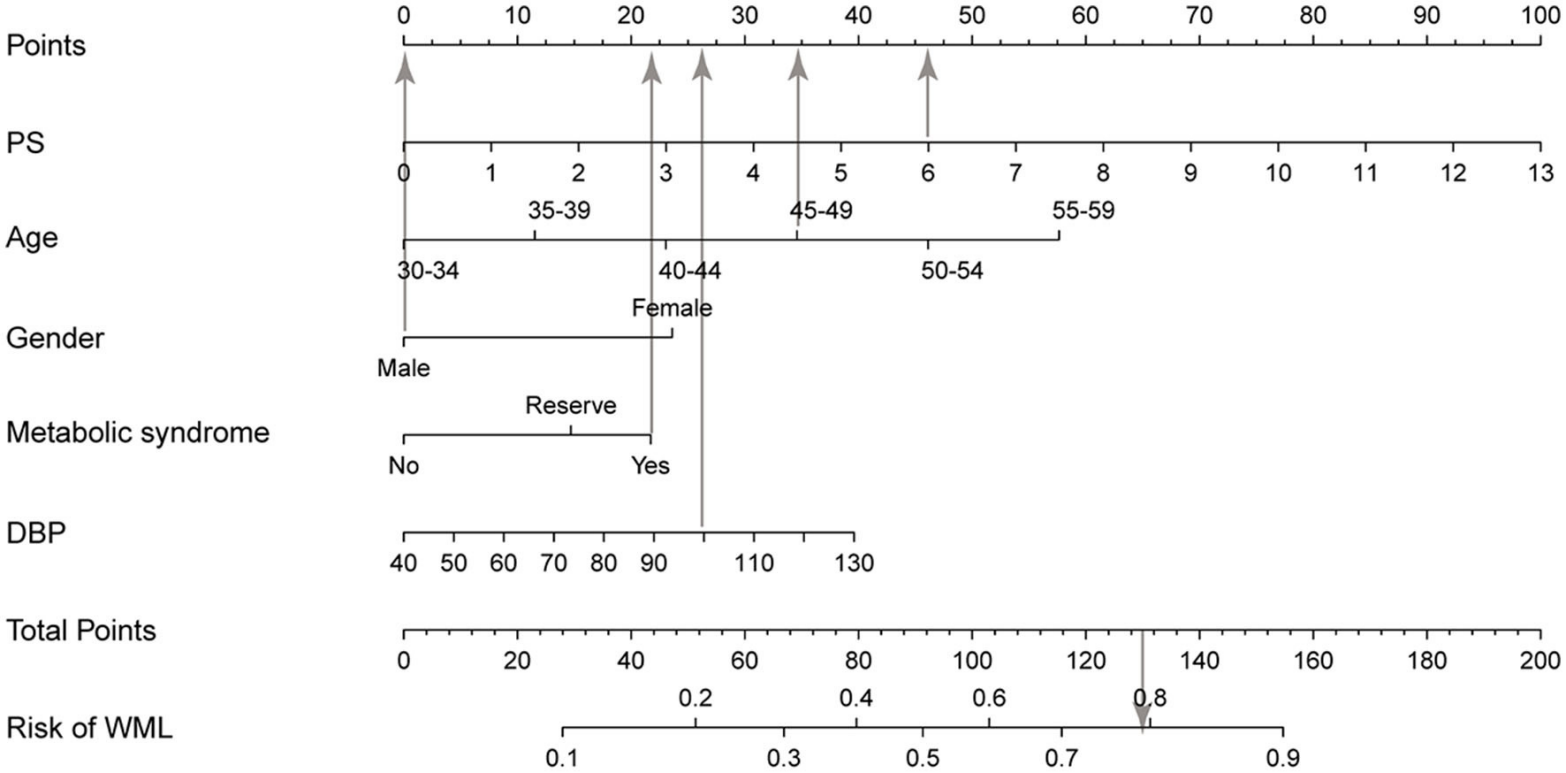
Nomogram for WML based on the training group. The nomogram for predicting WML in the study consisted of five variables. The total points were calculated as the sum of the score of the five variables included in the nomogram and finally converted the total point into a unique risk of WML with a percentage from 0 to 100%. PS, plaque score; DBP, diastolic blood pressure; WML, white matter lesion.

We developed a clinical prediction nomogram model (Figure 2) in the training group based on the five characteristics of the training group: age, DBP, PS, female gender, and metabolic syndrome, with a score range from 0 to 100 and then we summed each of them to calculate a total point, and we then converted the total point into a unique risk of WML with a percentage from 0 to 100%. It was suggested that the higher total point from the prediction nomogram indicated a higher risk of WML, and the lower total point indicated a lower likelihood of WML. Regarding the clinical application of the nomogram, we assumed that if a man aged 45 years, being in a state of metabolic syndrome, with a PS 6 and DBP measured 100 mmHg, then the corresponding score of WHL was 0+ 35 + 22 + 46 + 26, a total of 129 points; therefore, the risk of WHL in this person was nearly 0.8, and he was in a relatively high risk of WHL.

### Evaluation and calibration of the clinical prediction model

**Figure 3:**
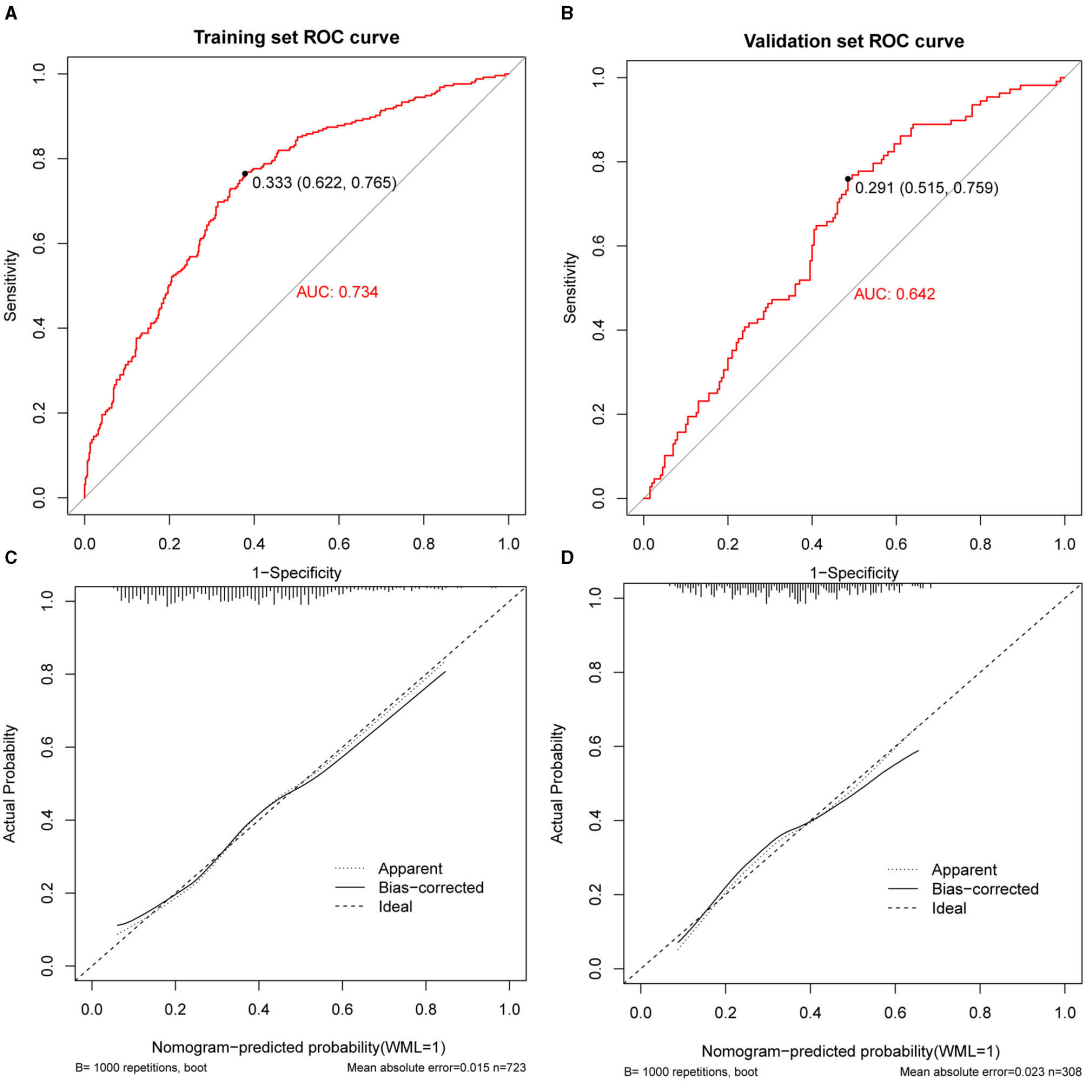
Receiver operating curve (ROC), area under the curve (AUC), and calibration curve in the training group and validation group. **(A, B)** Showed the receiver operating curve (ROC) and area under the curve (AUC) in the training group and validation group. **(C, D)** Showed calibration curves in the training group and validation group. The dashed line was the reference line where an ideal nomogram would lie. The dotted line was the performance of the nomogram, while the solid line corrected for any bias in the nomogram.

**Figure 4:**
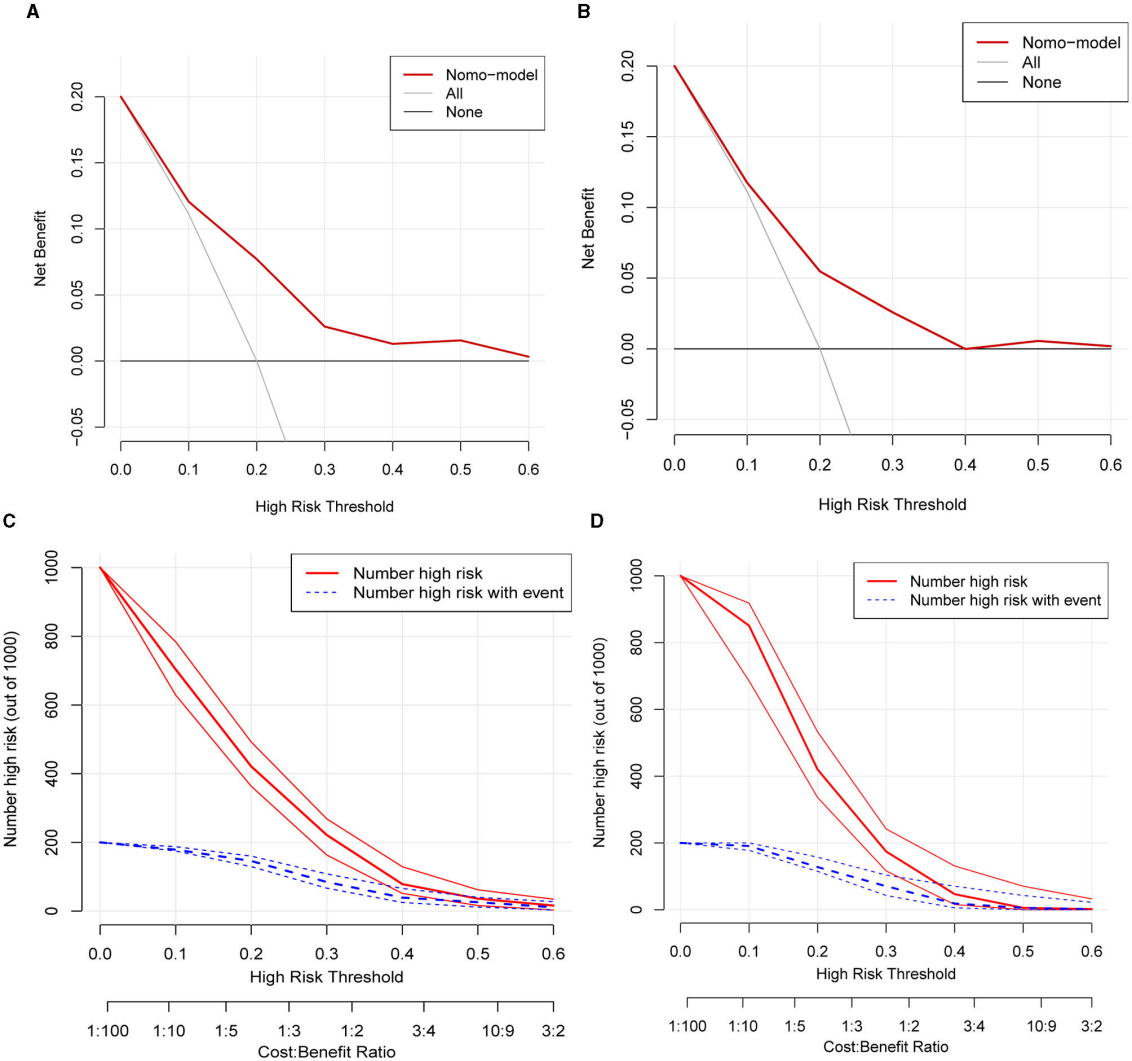
Decision curve and clinical impact curve of the training group and validation group. **(A, B)** Showed the decision curve of the training group and validation group. The red curve indicated the number of people classified as positive (high risk) by the model at each threshold probability, and the blue curve was the number of high risks with an event. The black line indicated that for extreme cases, the model predicted that all people have low-WML probability and the clinical net benefit was 0. The gray curve indicated that for extreme cases, the model predicted that all people have moderate- or high-WML probability and the clinical net benefit was the negative slope. The red line indicated that the model has clinical net benefit. The red line higher than the gray and black lines indicated that patients could benefit from the model. **(C, D)** Showed the clinical impact curve of the training group and validation group. The red curve indicated the number of people classified as positive (high risk) by the model at each threshold probability, and the blue curve was the number of high risks with an event.

Calibration curves were applied to evaluate the accuracy and consistency of the prediction model. ROC (Figures 3A, B) and calibration curve were plotted (Figures 3C, D) to test the discrimination and accuracy of the prediction model. The area under the curve (AUC) in the training group is 0.734 (95% CI 0.696–0.772), and the AUC in the validation group is 0.642 (95% CI 0.58–0.705), suggesting a good accuracy of the prediction model. The calibration curve in the training group and validation group showed that there was excellent consistency between the predicted WML probability and the actual WML probability. The decision curve (Figures 4A, B) and clinical impact curve (Figures 4C, D) in the training and validation group were drawn to test the clinical practical value, which showed a good clinical application value in the clinical prediction model.

## Discussion

We analyzed the data from a Japanese study to find out risk factors associated with WML in people <60 years old, and our results from multivariate regression analysis showed that the age (OR = 1.49, 95% CI: 1.31–1.70), diastolic blood pressure (OR = 1.02, 95% CI: 1.00–1.03), carotid plaque score (OR = 1.31, 95% CI: 1.14–1.50), female gender (OR = 2.27, 95% CI: 1.56–3.30), and metabolic syndrome (OR = 2.12, 95% CI: 1.22–3.70) were significantly associated with WML. Previous studies have revealed some risk factors associated with WML. Brown et al. (15) found that CSVD, age, hypertension, and current smoking were the potential causes of WML growth. Lin et al. (16) found that WML risk, WML occurrence, and WML progression were closely related to age, sex, hypertension, diabetes, smoking status, drinking habits, and the levels of HCY and LDL-C. Our study showed that with the increase in age, people were vulnerable to developing WML. In addition, female gender, higher DBP, higher carotid plaque score, and metabolic syndrome were independent risk factors for WML.

The carotid plaque score may more directly show the carotid artery atherosclerotic level (17), and some research studies confirmed that carotid atherosclerosis was related to the formation of WML (18, 19). We speculated carotid artery hemodynamic disorder caused by carotid disease was associated with cerebral hypoperfusion (20), and the microthrombus derived from unstable carotid plaques may aggravate the progress of WML (4). Previous studies have shown that age was an independent risk factor for WML. With the increase in age, the incidence and the volume of WML increased significantly (21). Our study showed that a considerable number of young people were found with WML and showed in an age-related manner. With the increase in age, atherosclerosis of the blood vessels resulted in a narrowing of the lumen and hardening of the blood vessel walls, which also occurred in small cerebral vessels, leading to cerebral small-vessel lesions, resulting in white matter damage (22). Our study showed that female patients were more prone to WML, which was similar to the PROSPER study (23). Moreover, the volume of WML increased faster in female patients, and the reason behind the sex difference remained unknown (24). Studies have shown that estrogen plays an important role in the brain, which can increase cerebral blood flow supply, resist oxidative stress, regulate synaptic growth, and inhibit neuronal apoptosis. The significant decrease in estrogen in postmenopausal women may lead to a decrease in brain blood supply and affect the nerve damage repair process, thus leading to brain damage in women (25, 26). Hypertension was closely associated with leukoencephalopathy, which was a significantly manageable risk factor for WML (27). Our results were familiar with the findings of Leung et al. (28) study, and their results showed that with the increase of average diastolic blood pressure, the severity of WMLs was also gradually increasing. People with long-term hypertension may suffer from vascular wall damage, resulting in arteriolar stenosis or microangiopathy, reducing the density and number of small vessels, resulting in insufficient arteriolar perfusion and small-vessel disease (29). Our results showed DBP, not SBP, was associated with WML. We speculated that the explanations might be that young- and middle-aged hypertension was mainly simple diastolic hypertension. In addition, DBP is an indicator of steady blood flow, and sustained diastolic hypertension may lead to negative effects on arterial remodeling or brain autoregulation (30). The visceral adipose tissue was mainly composed of omental and mesenteric adipose tissue, which could be divided into abdominal visceral fat and pelvic visceral fat, and its immune and metabolic activity was higher than that of subcutaneous adipose tissue. The visceral adipose tissue had a significant association with CSVD, including WML (31), and it was highly associated with metabolism-related diseases, which had attracted more and more attention from people. The visceral adipose tissue was a potential therapeutic target for the prevention of CSVD in future. Studies showed that the visceral adipose tissue had a close relationship with cardiovascular risk factors, such as blood pressure, elevated cholesterol levels, and diabetes, playing an important role in promoting the occurrence and development of WML (32–34). There is a controversy between diabetes and WML. Similar to other studies (35), our results showed that diabetes was not a risk factor for WML, and the potential mechanism needed further study.

Based on these risk factors, we constructed a clinical prediction model to predict the incidence of WML for people <60 years old. The nomogram for predicting WHL in the study consisted of five variables. The total points were calculated as the sum of the score of the five variables included in the nomogram, finally converted into an individual risk of WML expressed in percentage, ranging from 0 to 100%. The ROC, the calibration curve, and the clinical impact curve indicated that our prediction nomogram had good discrimination, accuracy, and clinical utility. There were several risk models based on these risk factors to predict the risk of WML. Shinkawa et al. (14) established a predictive model based on variables consisting of age, gender, PS, LDL, SBP, and administration of antihypertensive medication, which showed good accuracy to recognize risk probability for people with WML. Tully et al. (12) developed and validated a predictive model of WML for people > 65 years based on the risk factors of DBP, medication to reduce blood pressure, psychotropic drug use, dependence on IADL, forgetfulness, and arithmetic difficulties. However, these studies have not focused on these young- and middle-aged people, who are also a large group, with nearly 50% of WML. We identified the risk factors of WML in these young- and middle-aged people, and it is of great value to identify the high-risk population of WML in these people by constructing a prediction model. Meanwhile, these significant variables in our prediction model for each person could be easily obtained from health checkups, indicating the prediction model was highly practical. To the best of our knowledge, our prediction model was the first nomogram for young- and middle-aged people <60 years to predict the probability of WML.

For people at high risk of WML or with WML, we need to intervene in the risk factors to prevent the progression and aggravation of WML and the occurrence of stroke and cognitive impairment. In general, age and gender are both risk factors that could not be intervened, and the other three risk factors are also risk factors of stroke. For people with hypertension, especially those with high diastolic blood pressure, lifestyle changes are recommended, including weight loss, healthy dietary patterns (including reduced sodium and increased potassium intake), physical activity, and limited alcohol consumption. It is recommended that if blood pressure cannot be controlled after 6 months of intensive lifestyle intervention, early drug therapy should be considered (36). For people with carotid plaque, the dynamic re-examination of neck ultrasound is recommended. If these people are found with hyperlipidemia and carotid artery stenosis, it is recommended to change their lifestyle (more exercise and reduce fat intake) and take statins to reduce blood lipids and stabilize plaque (37). For people with metabolic syndrome, attention should be paid to lifestyle interventions, including maintaining an ideal body weight, exercising appropriately, changing the diet to reduce calorie intake, limiting salt, and reducing the intake of sugar-containing or sugar-substitute beverages (38).

### Limitations of the study

There were some limitations in our study. First, it was a single-center and retrospective study, and there was selective bias in the study. Therefore, multi-center and prospective studies were needed to provide more persuasive evidence to strengthen the accuracy and applicability of the prediction model. Second, there were no externally validated data in the prediction model; therefore, external data are still needed to further validate the predictive nomogram. Third, other important factors were not obtained in the Dryad Digital Repository database, including people's hobbies, occupations, and lifestyles, and moreover, radiology information was not available. These factors might also play an important role in WML. In addition, some other limitations should also be considered. There are many causes of white matter lesions, such as hereditary, inflammatory, infectious, and vascular. Especially for young patients, the causes may be diverse. However, our study cannot distinguish the causes of white matter lesions and the grading of white matter lesions is also important, but our study cannot grade. Since people over 60 years old are not included, it is not yet clear whether this model is suitable for those over 60 years old. Therefore, to comprehensively analyze the influencing factors of WML, it was necessary to conduct high-quality prospective studies.

## Conclusion

Age, diastolic blood pressure, carotid plaque score, female gender, and metabolic syndrome were risk factors in young- and middle-aged people <60 years old with WML, and the nomogram based on these risk factors showed good discrimination, accuracy, and clinical utility. Our study had important value for the early detection, early intervention, and early treatment of WML in young- and middle-aged people.

## Data availability statement

Publicly available datasets were analyzed in this study. This data can be found at: Dryad, https://datadryad.org/stash/dataset/doi:10.5061/dryad.73bh2q8.

## Ethics statement

Ethical review and approval was not required for the study on human participants in accordance with the local legislation and institutional requirements. Written informed consent from the patients/participants or patients/participants' legal guardian/next of kin was not required to participate in this study in accordance with the national legislation and the institutional requirements.

## Author contributions

RZ: Conceptualization, Data curation, Formal analysis, Investigation, Methodology, Writing—original draft. LP: Software, Writing—original draft, Data curation, Formal analysis, Methodology. QC: Formal analysis, Investigation, Software, Validation, Writing—original draft. YX: Formal analysis, Investigation, Methodology, Software, Writing—original draft. ZL: Conceptualization, Formal analysis, Investigation, Methodology, Software, Validation, Visualization, Writing—original draft. YL: Conceptualization, Project administration, Supervision, Visualization, Writing—review & editing.
